# VHH Antibodies: Reagents for Mycotoxin Detection in Food Products

**DOI:** 10.3390/s18020485

**Published:** 2018-02-06

**Authors:** Jia Wang, Hina Mukhtar, Lan Ma, Qian Pang, Xiaohong Wang

**Affiliations:** College of Food Science and Technology, Huazhong Agricultural University, Wuhan 430070, China; wangjia@mail.hzau.edu.cn (J.W.); hina@webmail.hzau.edu.cn (H.M.); mlan@mail.hzau.edu.cn (L.M.); qianp94@webmail.hzau.edu.cn (Q.P.)

**Keywords:** mycotoxin, VHH antibody, immunoassay

## Abstract

Mycotoxins are the toxic secondary metabolites produced by fungi and they are a worldwide public health concern. A VHH antibody (or nanobody) is the smallest antigen binding entity and is produced by heavy chain only antibodies. Compared with conventional antibodies, VHH antibodies overcome many pitfalls typically encountered in clinical therapeutics and immunodiagnostics. Likewise, VHH antibodies are particularly useful for monitoring mycotoxins in food and feedstuffs, as they are easily genetic engineered and have superior stability. In this review, we summarize the efforts to produce anti-mycotoxins VHH antibodies and associated assays, presenting VHH as a potential tool in mycotoxin analysis.

## 1. Introduction

**Figure 1:**
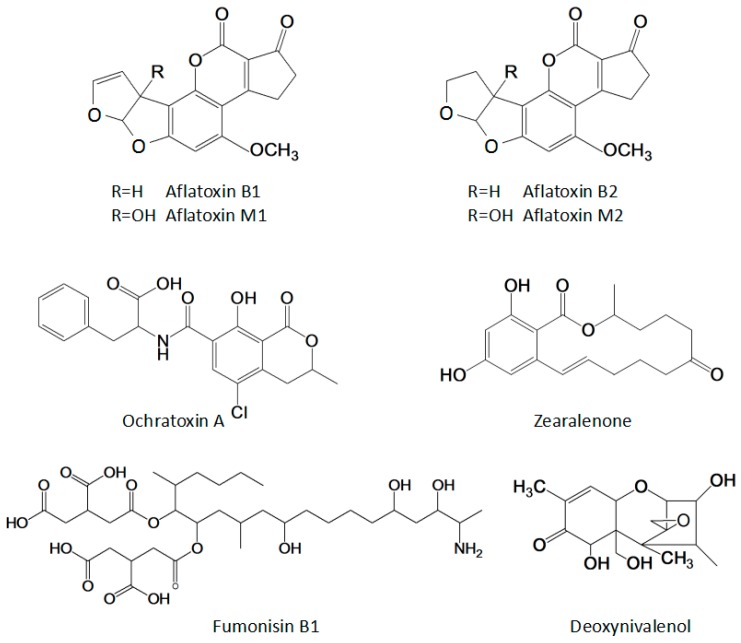
Molecular structures of the main mycotoxins.

Mycotoxins are the secondary metabolites naturally produced by fungi that colonize crops. Among more than 400 mycotoxins are discovered so far, Aflatoxins (AFTs), ochratoxins (OTs), fumonisins (FBs), deoxynivalenol (DON) and zearalenone (ZEN) are gaining attention due to their frequent occurrence and high toxicity in animals and humans (Figure 1) [1]. The maximum allowable limits for these mycotoxins have been set by national and international institutions and organizations [2,3]. The European Commission set maximum limits in unprocessed cereal for AFB1 at 2 µg/kg, OTA at 5 µg/kg, FB at 4000 µg/kg, DON at 1250 µg/kg and ZEN at 100 µg/kg [3]. Thus, sensitive analytical methods are used to identify and quantify them, including high-performance liquid chromatography (HPLC) with mass spectrometryc (MS) [4,5,6,7,8] and some nano-sensor technologies [9,10,11]. Among them, immunoassays based on specific interactions between antibodies and antigens are the most useful tools for rapidly monitoring of mycotoxins [12]. These assays have a number of advantages, including high selectivity toward the target analytes, greater than or comparable sensitivity to the instrumental methods and ease of operation and high-throughput screening ability [13] for mycotoxins in agricultural foodstuffs.

**Figure 2:**
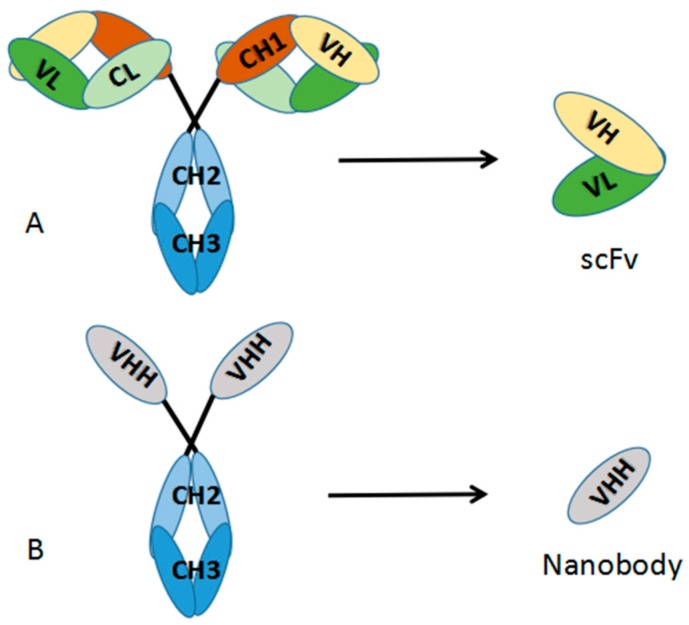
Schematic presentation of a (**A**) conventional antibody (IgG) and (**B**) heavy chain antibody, with their antigen-binding fragments (**A**) scFv and (**B**) VHH.

**Figure 3:**
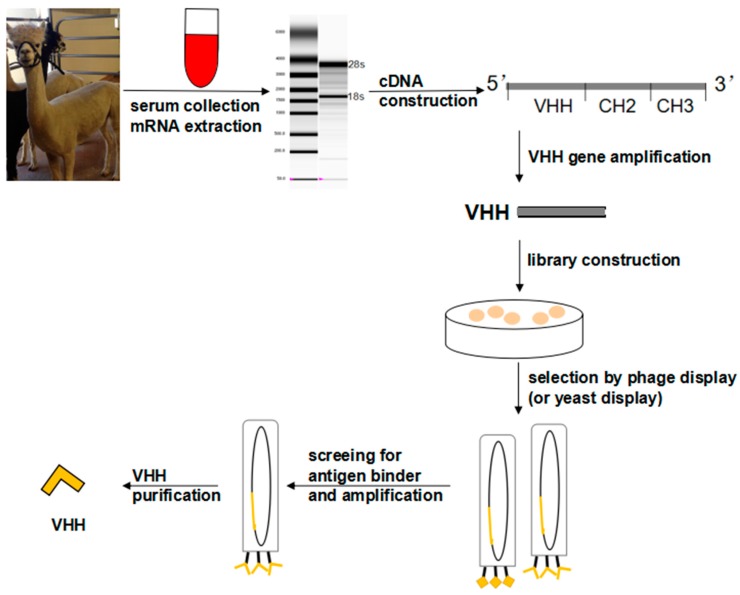
Overview of VHH generation from camelid.

Antibodies are the core reagents for immunoassays. In the serum of camelidae family and cartilaginous fish, heavy chain only antibodies (HcAbs) are existed devoid of light chains and lacking the CH1 domains (Figure 2) [14,15] The variable domain of HcAbs, which binds to specific antigens, is referred to as VHH. These unique antibody fragments are the smallest entities of the antigen-antibody interaction, so they are also called nanobodies [16]. VHH production is summarized in Figure 3 [17]. Compared with conventional antibodies (polyclonal antibodies [pAbs] and monoclonal antibodies [mAbs]), the VHHs are able to recognize the active sites of the antigen, which are inaccessible or cryptic for pAbs and mAbs [18] and they can be isolated and produced in expression systems (e.g., phage, yeast & ribosome selection systems) [19,20] with higher antibody yields. Compared with single-chain variable fragments antibodies (scFvs), the VHHs have greater or comparable sensitivity; can be produced in large quantities; are easily manipulated genetically; and have superior physicochemical stability and high refolding capacity [21,22], such as excellent solubility and resilience to organic solvents and high temperature [23,24,25]. The lower molecular weight (15 kD, 1/10 of conventional antibody), the single domain nature of VHHs and the evolutionary replacement of the light chain (VL) for antigen binding are compensated for by sequence heterogeneity in CDR regions and greater topological diversity in paratopes [26,27]. An naturally occurring disulfide bond between cysteine residues exits in VHHs and contributes largely to their conformational and thermal stabilities, without affecting antigen-antibody binding abilities [28]. Although VHH antibodies are also promising reagents for detecting small chemicals (MW < 1500 Da) in environmental monitoring [25] (e.g., picloram [29], triclocarban [30], 3-phenoxybenzoic acid [23], brominated diphenyl ether-47 [31] and tetrabromobisphenol A [24]), in the current review, we will focus only on the development and optimization of immunoassays for monitoring mycotoxins. In these cases, VHHs are used as antigen-binding entities, anti-idiotypic antibodies and recombinant proteins.

## 2. Anti-Hapten VHH-Based Immunoassay

**Figure 4:**
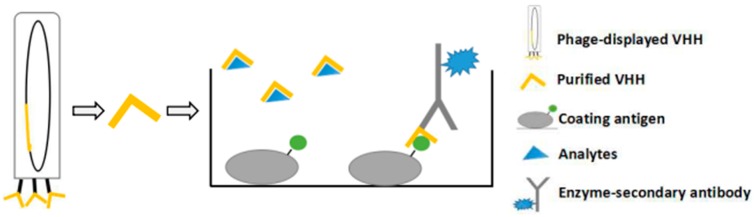
VHH production and anti-hapten VHH-based immunoassay. Coating antigen and analytes competitively bind to primary antibody, followed by the enzyme-secondary antibody and enzyme substrates. A weaker signal indicates a higher analyte concentration.

Generally, immunoassays that detect mycotoxins are based on a competitive assay, typically in direct or indirect format. Take the indirect format as an example, the solid phase is sensitized by coating antigen, followed by incubating with test analytes and anti-hapten antibodies. The antibodies bound to the coating antigen are measured and this determines the amount of analyte present [32] (Figure 4). The VHHs, function as binding with analytes preferred to be isolated mostly from an antigen-immunized VHH library. The anti-hapten VHHs are selected from phage displayed VHH fragment libraries, with a sequential competition strategy [33]. The immunogen is synthesized chemically, attached to a carrier protein and injected subcutaneously; three to five injections are used to boost the immune reaction. RNA is isolated from the blood after the last immunization. The first strand of cDNA was synthesized and the VHH domain is amplified and ligated into the expression system to construct the library [34]. Phage display technology is a preferable expression system, with the foreign nucleotide sequence encoded by the virion and foreign peptides or proteins expressed on its surface [35]. VHHs can be easily isolated, enriched, expressed and purified from phage display VHH library (Figure 4) [36]. The VHHs can be expressed and purified in columns. With phage display technology, the displayed VHH and phage DNA can be used directly as the detection antibody and DNA template in phage display-mediated immune-polymerase chain reaction (PD-IPCR), a technique with ultra-sensitivity. In PD-IPCR, the SYBR premix is added into each reaction and the fluorescent signal is detected during PCR amplification. The procedure is the same as the indirect immunoassay, except the VHH phage supernatant is used in place of the primary antibody.

A phage displayed OTA-specific VHH, obtained from a phage displayed VHH library with OTA protein as immunogen in alpaca, was employed to develop a competitive indirect ELISA [37,38]. Then, a competitive real-time PD-IPCR for OTA was developed. The detection level of this method was 3.7 pg/L, with a linear range of 0.01–1000 pg/mL [39]. A His-tag can help anti-OTA VHH be purified on a nickel sepharose column with high purity. With the competing antigen OTA-BSA on a polyvinylidene fluoride membrane, a VHH-based dot ELISA was developed and the color intensity of the test squares was determined either visually or by scanning and quantitative analysis with densitometry. The cut-off level of this method was 5 µg/kg visually for OTA, with weak cross-reactivity with OTB and no cross-reactivity with DON, AFB1 and ZEN [40].

Anti-AFB1 VHH was also displayed in phage and purified by nickel sepharose column [41]. The magnetic-bead-carrying poly (acrylic acid) brushes (MB@PAA) were fabricated with anti-AFB1 VHH for improving AFB1 adsorption capacity. The MB@PAA@VHH exhibited good AFB1 adsorption (0.23 mg/g) and reusability with AFB1-spiked corn samples [42]. VHHs show much better tolerance to high temperature and organic solvents than mAbs. During exposure to 95 °C for 5 min, mAbs lost their antigen-binding abilities, while VHHs did not [41,43]. After incubation at 85 °C for 1 h, the anti-AFB1 VHHs Nb26 and Nb28 still retained about 70% and 40% of their binding activities, respectively [41]. VHHs are resistant to acetone, acetonitrile and methanol, which are the preferred solvents of highly lipophilic analytes in sample extraction. Both Nb26 and Nb28 retained 100% of their binding abilities in 80% of methanol, whereas a representative mAb lost 50% of its binding ability under the same conditions. Moreover, the mAb lost all of its AFB1-binding ability in the presence of 60% acetonitrile [41].

## 3. Anti-Idiotypic VHH Immunoassay

Anti-idiotypic antibodies, induced by paratopes of a primary antibody, are used as the secondary antibody against “idiotypic” or idiotopes [44]. This antibody competes with target analytes to bind with the same region of a primary antibody. Anti-idiotypic antibody is an exciting reagent because (1) it represents the “internal image” of the original antigen [45]; (2) it serves as a surrogate for the original antigen [46]; and (3) it competes with the original antigen for the primary antibody. In contrast to the VHH selection, anti-idiotypic VHHs are usually isolated from the library constructed by primary antibody immunization and eluted with the free antigen. Combined with the VHHs, anti- idiotypic VHH is a promising alternative to using the highly toxic mycotoxins in immunoassay.

### 3.1. Anti-Idiotypic VHH as a Substituent for Analytes

Due to the toxicity of mycotoxins and their hazardous effects to operator and environment, surrogate analytes were introduced to generate a standard calibration curve and assist in calculating the analyte concentration, based on the equation from the surrogate analyte [47]. In competitive immunoassays, anti-idiotypic antibodies (non-toxic reagents) were able to bind to the primary antibody and mimic the configuration with analytes; thus, they can be used as a surrogate analytes to develop an immunoassay without mycotoxins [48]. Anti-idiotypic VHH 2-5 against AFB1 was developed by immunizing an alpaca with anti-AFB1 mAb 1C11. VHH 2-5 showed almost the same binding abilities to mAb 1C11 as the homologous scFv 2G7 from 1C11, which indicated that VHH2-5 (by some key amino acid residues) might bind to AFB1 in the same way with that scFv 2G7 bound to the AFB1 [46]. With AFB1-BSA coated on the microplate and series dilution of VHH 2-5 incubation, the calibration curve of surrogate VHH also fit a four-parameter logistic equation (R^2^ = 0.9969). The same percentage of inhibition by VHH 2-5 and AFB1 were compared and it reflected the high correlation between their concentrations (R^2^ = 0.9985). Therefore, VHH 2-5 can be used as a surrogate analyte for AFB1 to develop a toxin-free ELISA. After a simple extraction with methanol/H_2_O (3:1, *v/v*), the VHH 2-5 concentration was determined first and then converted to AFB1 concentration with a linear equation. The assay had a comparable limit of detection and correlated well (R^2^ = 0.988) with the HPLC method in 20 contaminated peanut samples [49].

**Figure 5:**
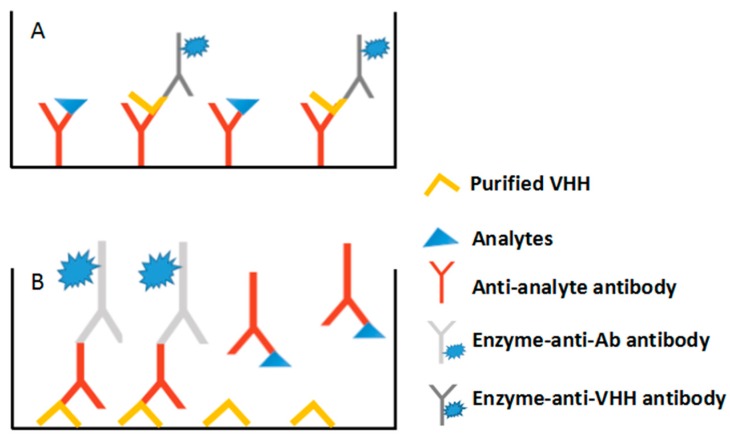
Anti-idiotypic VHH-based Immunoassay. (**A**) Anti-idiotypic VHH functions as substitute for analytes. (**B**) Anti-idiotypic VHH functions as a mimic competing antigen.

Because the anti-idiotypic antibody can compete with analytes for binding to the primary antibody, it can be used as a substitute for analytes (Figure 5A). Thus, an indirect competitive phage ELISA was developed for citrinin, a secondary metabolite found in food and feeds and a by-product of fermentation by the red yeast *Monascus purpureus* [50]. Compared with conventional anti-idiotypic antibodies, the anti-idiotypic VHHs can serve as both the detection antigens and DNA templates, therefore they can be used for immune-PCR (IPCR) with high sensitivity. A non-toxin quantitative IPCR assay was then developed for detecting OTA in agricultural products, with a limit of detection (LOD) of 4.17 pg/mL [51]. The M13 phage containing AFB1 anti-idiotypic VHH was used as a reagent for accurately quantifying AFB1 in corn, rice, peanut and feedstuffs. The real-time IPCR method brought higher sensitivity (4-fold increase over phage ELISA) and a wider linear range [52].

Anti-idiotypic VHH has also been used as a non-toxic surrogate for ZEN for a “green” immunoassay. One DNA sequence was obtained after four rounds of biopanning. Coating with anti-ZEN mAb, the phage ELISA for ZEN was developed; it gives an IC_50_ of 0.25 ng/mL and a linear range of 0.11–0.55 ng/mL. The PD-IPCR for ZEN gave a detection limit of 6.5 pg/mL, a 12-fold improvement over phage ELISA [53]. 

Anti-idiotypic VHHs also represent the benefits of VHHs, showing high tolerance to methanol and thermostability [46]. Polyclonal anti-idiotype AFM1 antibody lost all of its primary antibody-binding ability after 5 min incubation at 80°C; whereas, VHH retained 50% of its activity after 20 min incubation [49].

### 3.2. Anti-Idiotypic VHH as a Mimic Competing Antigen in Immunoassay

Competitive immunoassays is the typical method used to detect mycotoxins in contaminated food products [54]. The coating antigens, usually obtained by coupling the mycotoxin with a carrier protein via chemical synthesis, are commonly used as the competing compound against primary antibodies [54]. Heterologous site systems, coupling carrier proteins with haptens as the immunogen and coating antigen from different positions [55] or different length in spacer-arm [56], are effective tools for improving the sensitivity and specificity of immunoassay. Unfortunately, the chemical synthesis of coating antigen inevitably exposes operators to these toxic analytes. Besides, the extensive modification and bridge group interference may make the conjugates less efficient or produce some unwanted cross-reactions [57]. Phage displayed peptide, isolated from the phage-displayed peptide library, is also a reagent to replace the chemical conjugated coating antigen. Although the peptide recognizes the primary antibody binding site and shows competition with the analytes for binding, it is rarely used as the coating antigen directly. Conjugating with carrier protein and fusion with binding protein [58] are the two options for using peptides as coating antigens. However, the molecular ratio of the peptide and carrier protein to the expression system in vivo may decrease the binding abilities of peptides to some extent. Due to the monovalence and mimicry of the toxins binding to primary antibodies, VHH can serve as the coating antigen [59] and potentially replace the conventional coating antigens (Figure 5B).

As a model, VHH was used to develop an immunoassay for DON. VHHs (N-28 and N-31) were isolated from a naïve phage display library through coating with anti-DON mAb and were used as coating antigen mimics in a heterologous immunoassay. Compared with chemically synthesized coating antigen (DON-BSA), the N-28 and N-31–based assays enhanced the assay sensitivity by 18-fold and 8-fold, respectively. Molecular modeling and alanine-scanning mutagenesis revealed that the residues Thr 102-Ser 106 of N-28 contributed to the binding with mAbs [60]. To improve the sensitivity of the assay further and to understand the relationship between anti-idiotypic VHH and mAb, site-saturation mutagenesis was applied to study the key residue Thr 102 [61]. An immunoassay with the mutant N28-T102Y gave an IC_50_ of 24.49 ng/mL, with sensitivity 3.2 times lower than N-28. Compared with the homology modeling of N-28, the mutant N28-T102Y had a larger side chain at position 102, shifting the binding site to the edge of the binding pocket, decreasing binding activity with anti-DON scFv [62].

As with FB1 and citrinin analysis, anti-idiotypic VHHs are an excellent alternative to chemosynthetic coating antigen. The anti-idiotypic VHH Ab2β Nb anti-FB1 was obtained from a naïve alpaca nanobody library. The Ab2β Nb was used as an alternative to chemosynthetic FB1-BSA conjugates, increasing the assay sensitivity by ~20-fold. The equilibrium dissociation constant (K_D_) measured for Ab2β Nb: anti-FB1 mAb was 164.6nM, which was 270 times higher than the K_D_ of FB1-BSA: anti-FB1 mAb (K_D_ = 0.62 nM) [63]. The weaker affinity indicates that less coating antigen participated in the competitive immunoassay [64,65]. As a result, the sensitivity of the assay was improved. Similarly, one β-type VHH was selected as a coating antigen from a naïve phage displayed VHH library and used to develop an immunoassay for citrinin detection; it produced an IC_50_ that was twice as good as that of citrinin-ovalbumin conjugates-based ELISA [66].

The anti-idiotypic VHH can be used as a signal-amplification carrier for an indirect competitive phage ELISA, showing comparable sensitivity with conventional chemosynthetic citrinin conjugate-based immunoassays [67,68]. Anti-idiotypic nanobodies can also be conjugated with the Eu/Tu (III) nanosphere as a probe to establish the competitive time-resolved strip methods for AFB1 and ZEN detection simultaneously. In this case, the probes were applied to a nitrocellulose membrane as capture antigens, with anti-AFB1 mAb and anti-ZEN mAb as detectors, providing a quantitative relationship ranging from 0.13 to 4.54 ng/mL for AFB1 and 0.20 to 2.77 ng/mL for ZEN [69].

## 4. VHH-Based Recombinant Proteins

VHHs can be chemically modified for site-specific immobilization to a transducer surface [70,71] or branched to nanoparticles and magnetic nanoparticles [42]. VHHs have great potential as bio recognition elements in the construction of immunosensors for mycotoxins [36]. Although, mAb- and pAb-based biosensors are traditionally used, recombinant antibody (rAb)-based biosensors have been reported to detect sub-regulatory levels of fungal (mycotoxins), marine (phycotoxins) and aquatic bio toxins in a wide range of food and environmental matrices. Due to this great potential for rAb technology, we can predict the inherent advantages of engineered rAb to provide the next generation of ultra-high performance binding reagents for rapid and specific toxin detection [53]. VHHs, the smallest specific-binding entities, can be genetically manipulated for a variety of applications [72]. To simplify immunoassay protocols, fluorescent proteins [72] or enzymes, such as alkaline phosphatase (AP), can be fused with VHHs for one-step immunoassay [73]. Besides, avoiding random chemical conjugation and the use of secondary antibodies, VHHs with fused proteins enlarge the paratopes and thus improve the affinities of VHHs due to the homodimeric nature of AP [74]. Anti-idiotypic nanobodies can also be fused with AP and expressed in E. *coli*, as demonstrated by the one-step detection of FB1 [75]. The anti-idiotypic VHH-AP retained both the enzymatic activity of AP and the ability to bind with primary antibody. The K_D_ was 65.5 nM for Ab2b-Nb-AP: anti-FB1 mAb, which was half as large as that of Ab2b-Nb: anti-FB1 mAb (164.6 nM). In other words, the affinity of Ab2b-Nb-AP for anti-FB1 mAb was twice as large as for VHH, showing that the fused VHH-AP can enhance the binding affinity [76]. The colorimetric ELISA and chemiluminescence ELISA were developed with the “clonable” anti-idiotypic VHH-AP homogeneous probe for FB1, with an IC_50_ of 2.69 ng/mL and 0.12 ng/mL, respectively [75]. A direct competitive fluorescence enzyme immunoassay was performed for OTA detection, with IC_50_ and detection limit of 0.13 and 0.04 ng/mL, respectively [77]. Finally, 15-acetyl-deoxynivalenol (15-AcDON) was one of the mostly commonly DON acetylated metabolites in contaminant maize and small-grain cereals [78]. A monomer VHH was isolated from an antigen-immunized library and a VHH pentamer was formed by fusing the monomer DNA to the verotoxin B subunit in the pVT2 expression vector. The pentamer fragment showed structural flexibility for binding to antigen and pentamerization increased the affinity of the VHH for 15-AcDON [79]. Both the monomer and pentamer fragments can bind to 15-AcDON, with IC_50_ values of 1.24 and 0.5 µM respectively (determined with a competitive fluorescence polarization assay) [80].

## 5. Conclusions

Antigen-specific antibodies (also called recognition elements) are the most important and challenging components for mycotoxins immunoassays. VHHs, as an emerging reagent for monitoring hazardous mycotoxins in food (Table 1), have many advantages, including high solubility, ease of genetic manipulation and resistance to high temperature and organic solvents, despite the fact that they can be expensive to produce and their efficacy can be affected by the varied response of the animals and primer bias. VHHs are also very useful in crystallizing conformationally and the interaction between VHHs and the target [81]. However, to our knowledge, no crystal structure of an anti-mycotoxin VHHs against mycotoxin has been reported. Once we identify the VHH structures, it would be useful to identify the amino acid that influence binding affinity and carry out a mutagenesis study to enhance the assay sensitivity. In summary, VHHs are promising tools for detecting mycotoxins in food products.

## Figures and Tables

**Table 1:** VHH generation and applications for mycotoxin detection.

**Anti-Hapten VHH**				
**Analyte**	**VHH Library**	**Immunoassays**	**Sensitivity, IC_50_**	**Linear Range (LOD)**
OTA	Immunogen OTA-KLH	Indirect competitive ELISA [37]	12 ng/mL	8–20 ng/mL (6 ng/mL)
	Immunogen OTA-KLH	Indirect competitive ELISA [38]	0.64 ng/mL	0.27–1.47 ng/mL
	Immunogen OTA-KLH	Competitive real time PD-IPCR [39]		0.01–1000 pg/mL (3.7 pg/L)
	Immunogen OTA-KLH	VHH-based dot ELISA [40]		(5 µg/kg)
AFB1	Immunogen AFB1-BSA	Indirect competitive ELISA [41]	0.754 ng/mL	0.117–5.676 ng/mL (0.05 ng/mL)
**Anti-Idiotypic VHH**				
AFB1	Immunogen: anti -AFB1 mAb 1C11	Indirect competitive ELISA [49]		0.018–0.079 ng/mL (0.015 ng/mL)
	Immunogen: anti -AFB1 mAb 1C11	VHH ELISA [46]	0.16 ng/mL	
	Immunogen: anti -AFB1 mAb 1C11	Real-Time Immuno-PCR [52]		(0.02 ng/mL)
OTA	Naïve VHH phage display library	Immuno-PCR [51]	300 pg/mL	(4.19 pg/mL)
ZEN	Naïve VHH phage display library	Indirect competitive phage ELISA [53]	0.257 ng/mL	0.11–0.55 ng/mL (0.08 ng/mL)
	Naïve VHH phage display library	Phage display mediated IPCR [53]		0.01–100 ng/mL (6.5 pg/mL)
DON	Naïve VHH phage display library	Indirect competitive immunoassay [60]	8.77 ng/mL	2.18–62.25 ng mL (1.16 ng/mL)
	Naïve VHH phage display library	Competitive phage ELISA [62]	24.49 ng/mL	9.51–180.15 ng/mL
citrinin	Naïve VHH phage display library	Phage ELISA [50]	10.9 µg/kg	2.5–100.0 µg/kg
	Naïve VHH phage display library	VHH ELISA [66]	44.6 ng/mL	5.0–300.0 ng/mL
FB	Naïve VHH phage display library	Competitive ELISA [63]	0.95 ng/mL	0.27–5.92 ng/mL (0.15 ng/mL)
AFB1 ZEN	Immunized alpaca library	Time-resolved fluorescence immunochromatographic assay [69]	0.46 and 0.86 ng/mL for AFB1 and ZEN	AFB1: 0.13–4.54 ng/mL (0.05 ng/mL) ZEN: 0.20–2.77 ng/mL (0.07 ng/mL)
